# Hearing Loss in Leucine-Rich Glioma-Inactivated 1 Encephalitis: Cochlear Implantation Considerations

**DOI:** 10.7759/cureus.89296

**Published:** 2025-08-03

**Authors:** Nawfal Saleem, Mackenzie Robbins, Jacob Foster, Benjamin Kelley

**Affiliations:** 1 Otolaryngology, Lake Erie College of Osteopathic Medicine - Bradenton (LECOM-Bradenton), Bradenton, USA; 2 Pathology, Lake Erie College of Osteopathic Medicine - Bradenton (LECOM-Bradenton), Bradenton, USA; 3 Otolaryngology, University of Texas Medical Branch Otolaryngology Residency Program, Galveston, USA; 4 Otolaryngology - Head and Neck Surgery, Manatee Memorial Hospital, Bradenton, USA

**Keywords:** autoimmune encephalitis, cochlear implant, hearing loss, lgi-1 autoimmune encephalitis, sensorineural hearing loss, snhl

## Abstract

Leucine-rich glioma-inactivated 1 (LGI-1) antibody-associated autoimmune encephalitis is a rare neurologic disorder primarily presenting with memory impairment, behavioral changes, and seizures. Auditory symptoms, particularly sensorineural hearing loss (SNHL), are uncommon but may occur due to LGI-1 expression in auditory pathways.

A 78-year-old male with a prior diagnosis of LGI-1 autoimmune encephalitis presented with progressive bilateral SNHL and frequent falls. His audiogram revealed significant deficits in high frequencies and impaired speech recognition without evidence of middle ear pathology, affecting quality of life. Neuroimaging showed chronic white matter lesions consistent with LGI-1 encephalitis. Cochlear implantation was discussed as a potential rehabilitative treatment.

This case underscores SNHL as a potentially significant yet rare manifestation of LGI-1 antibody-associated autoimmune encephalitis. The patient's profound hearing loss suggests an autoimmune mechanism affecting both central auditory and peripheral cochlear pathways. Cochlear implantation, although not widely studied in autoimmune-mediated auditory disorders or trialed in this case, emerged as a promising option for rehabilitation, necessitating further investigation into its efficacy and safety.

## Introduction

Leucine-rich glioma-inactivated 1 (LGI-1) antibody-associated autoimmune encephalitis is a rare neurologic disorder characterized by subacute onset of neuropsychiatric symptoms, including memory impairment, behavioral changes, and seizures, most notably faciobrachial dystonic seizures. MRI frequently reveals abnormalities in the medial temporal lobes, and CSF may show mild inflammatory changes. Hyponatremia is also common in this syndrome [1-3].

LGI-1 autoimmune encephalitis belongs to a group of autoimmune encephalitides that target proteins associated with the voltage-gated potassium channel (VGKC) complex. Among these, contactin-associated protein-like 2 (CASPR2) is another extracellular protein that is associated and linked with the VGKC complex. The pathogenic antibodies in these syndromes are directed against LGI-1 or CASPR2 and not the potassium channel itself. Patients with positive LGI-1 antibodies often present with limbic encephalitis, whereas CASPR2 antibodies are linked to Morvan syndrome, neuromyotonia, and involvement of both the central and peripheral nervous systems. In fact, the term "VGKC complex antibodies” is now considered obsolete in clinical practice, since only LGI-1 and CASPR2 antibodies have well-defined clinical relevance and VGKC antibody positivity without LGI-1 or CASPR2 specificity is not reliably associated with a specific clinical syndrome and should not be used to guide diagnosis or management [4,5].

Autoimmune encephalitis is increasingly recognized as a cause of subacute neurocognitive decline, seizures, and psychiatric symptoms. Among these, antibodies targeting components of the VGKC complex, most notably LGI-1 and contactin-associated protein-like 2 (CASPR2), are associated with distinct clinical syndromes. Detection of LGI-1 antibodies has become a reliable diagnostic marker for limbic encephalitis, often presenting with cognitive dysfunction, faciobrachial dystonic seizures, and hyponatremia.

While auditory symptoms such as hearing loss are not commonly reported in LGI-1 encephalitis, the expanding spectrum of autoimmune neurologic disorders increasingly includes sensory deficits. Notably, LGI-1 is expressed not only in the central nervous system but also in the peripheral auditory system, particularly at the synaptic junctions where it plays a role in stabilizing synaptic transmission, which may explain the potential for auditory involvement in some cases [6,7]. 

The clinical presentation of LGI-1 antibody-associated encephalitis is heterogeneous, often leading to misdiagnosis as other neurological or psychiatric disorders. For instance, a case report detailed the case of a 46-year-old male initially misdiagnosed with schizophrenia due to presenting symptoms of depressive mood, personality changes, and visual hallucinations, which later progressed to include memory impairment and seizures [1]. Although not initially recognized, such presentations underscore the diagnostic heterogeneity of LGI-1 encephalitis and raise the possibility that under-recognized sensory symptoms, including hearing loss, may also be overlooked during early evaluations. Neuroimaging typically reveals bilateral temporal lobe lesions, and positive serum and CSF tests for LGI-1 antibodies confirm the diagnosis [1,2].

Treatment primarily involves immunotherapy, including corticosteroids and intravenous immunoglobulin (IVIG), which have shown significant efficacy in improving clinical outcomes. Early recognition and treatment are crucial to prevent long-term cognitive deficits and improve patient prognosis [6,7].

We present a rare case of LGI-1 encephalitis with concomitant sensorineural hearing loss (SNHL) and discuss the potential role of cochlear implantation (CI) as a rehabilitative option. To our knowledge, this is among the first reported cases to consider CI in the setting of autoimmune encephalitis mediated by LGI-1 antibodies. This case highlights the importance of considering autoimmune etiologies in sudden hearing loss and the need for further research into auditory involvement in paraneoplastic and autoimmune neurologic syndromes.

## Case presentation

A 78-year-old Caucasian male presented to our otolaryngology clinic with a chief complaint of progressive hearing loss and falls. This was suspected to be multifactorial in nature, likely related to his central neurologic impairment, given the presence of LGI-1 encephalitis. However, physical examination did not reveal signs of vertigo, nystagmus, or other otologic findings, making a peripheral vestibular cause less likely. His medical history is significant for LGI-1 autoimmune encephalitis, initially diagnosed in 2016. Since the onset of his illness, he has developed notable memory impairment and SNHL. The patient denied any other associated otologic symptoms, including tinnitus, vertigo, otalgia, or otorrhea. 

His past medical history was unremarkable, and there was no pertinent family history of hearing loss. He reported childhood noise exposure while sharpening farm tools, but no significant occupational exposure or history of ototoxic medication use. At presentation, he was actively maintained on immunosuppressive therapy for long-term management of his autoimmune condition, although specific agents were not available in the medical record.

**Figure 1:**
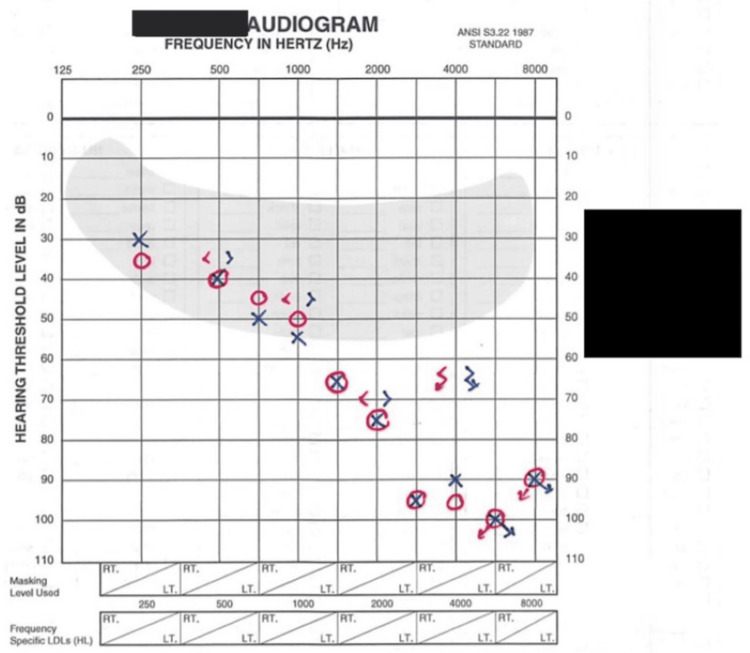
Audiogram Audiogram demonstrating bilateral HFSNHL. Pure-tone thresholds are shown as red circles (right ear air conduction), red arrows (right ear bone conduction), blue Xs (left ear air conduction), and blue arrows (left ear bone conduction). Findings reveal profound hearing loss, particularly at frequencies above 2000 Hz, affecting both air and bone conduction thresholds. HFSNHL, high-frequency sensorineural hearing loss; LDL, loudness discomfort level; HL, hearing level; RT, right ear testing values; LT, left ear testing values.

Otoscopy revealed clear external auditory canals, and there were no signs of middle ear pathology bilaterally. Figure 1 shows an audiometric evaluation, which reveals profound bilateral high-frequency sensorineural hearing loss (HFSNHL) consistent with cochlear or retro-cochlear involvement. Patient speech recognition is also significantly decreased, as shown in Table 1. 

**Table 1:** Speech test and discrimination test results This table summarizes the patient’s audiometric performance, including pure-tone averages, speech reception thresholds, loudness discomfort levels, most comfortable listening levels, and word recognition scores for the right ear, left ear, and binaurally. The results highlight fair to poor speech discrimination secondary to his hearing loss. HTL, hearing threshold level; SRT, speech reception threshold; LDL, loudness discomfort level; MCL, most comfortable level; MCC, most comfortable condition.

Ear	Speech Test Results						Discrimination Test Results	
	Average (HTL) Pure-Tone	SRT (HTL)	LDL (HTL)		MCL (HTL)		Word Recognition % Correct	Presentation Level
Right	55	65	115+		90		68%	MCC
Left	57	70	115+		95		64%	MCC
Binaural	n/a	n/a	Left 105	Right 100	Left 85	Right 80	72%	MCC

**Figure 2:**
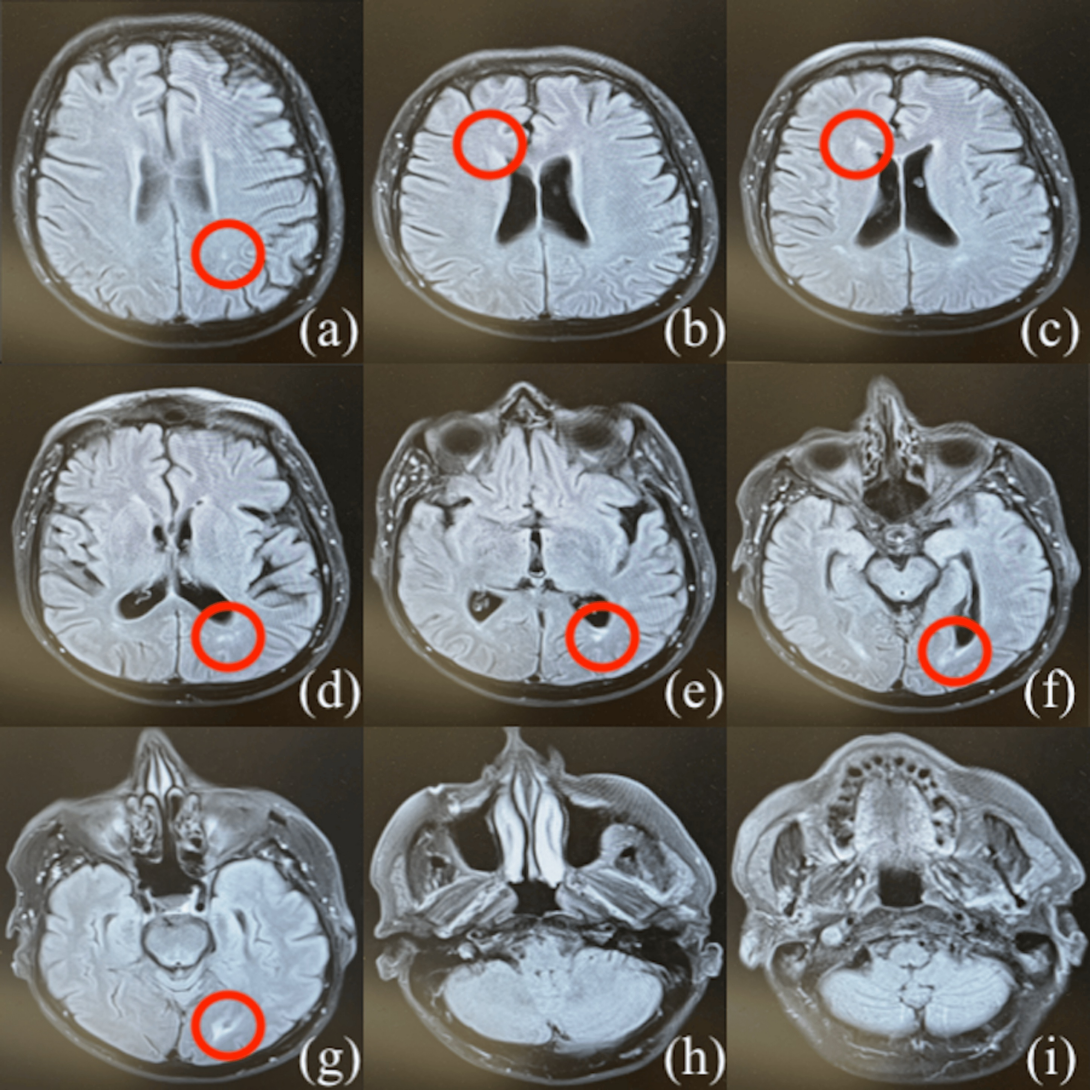
T2-weighted axial-view MRI images of the patient's brain. MRI images demonstrate progressive slices from superior to inferior. Red circles highlight regions of abnormal white matter lesions consistent with inflammatory involvement affecting cortical and subcortical structures. The sequence illustrates the extent and distribution of lesions, consistent with LGI-1 autoimmune encephalitis. LGI-1, leucine-rich glioma-inactivated 1.

MRI demonstrated multiple white matter lesions in the subcortical and periventricular regions, consistent with his prior diagnosis of LGI-1 autoimmune encephalitis (Figure 2). The lesions showed no acute changes and have remained stable since his initial diagnosis in 2016.

There was no evidence of middle ear pathology on otoscopic examination or tympanometry. Given the degree of his SNHL and impact on speech recognition, the CI was considered and discussed as a potential rehabilitative option. The CI was deferred for now as the patient opted to trial an external hearing aid before pursuing more invasive measures. The authors intend to update the case study should the patient decide to undergo a CI in the future.

## Discussion

This case emphasizes the clinical spectrum of LGI-1 antibody-associated autoimmune encephalitis by highlighting SNHL as a significant yet uncommon complication. While auditory involvement is not typically recognized as a common manifestation in LGI-1 encephalitis, this patient's progressive bilateral high-frequency SNHL, in the absence of other identifiable otologic etiologies, suggests a potential autoimmune mechanism involving bilateral cochlear and/or peripheral retro-cochlear pathways, consistent with LGI-1 expression in both central and peripheral auditory synaptic regions [3].

LGI-1 antibodies target proteins critical for normal synaptic function, potentially leading to impaired neural transmission in auditory pathways. Given the LGI-1 expression in both central nervous structures and the cochlea, autoimmune-mediated damage can plausibly affect auditory processing and lead to the sensorineural deficits observed in this patient [6]. Similar presentations in autoimmune disorders suggest that the underlying immunological mechanisms could involve direct antibody-mediated effects, secondary inflammatory changes, or a combination of both. While additional testing for CASPR2 or VGKC complex antibodies may have been performed, documentation of these results was unavailable. However, the detection of LGI-1 antibodies alone is clinically significant and sufficient to support the diagnosis, as VGKC complex antibody positivity without LGI-1 or CASPR2 specificity is no longer diagnostic [4,5].

The patient's SNHL has impaired his speech recognition, underscoring the critical impact of auditory dysfunction on quality of life. In light of limited auditory rehabilitation options available, CI emerged as a potential treatment. While CI is widely used for SNHL, its application in autoimmune-mediated auditory dysfunction remains relatively unexplored. The potential efficacy of CI in this context warrants further investigation, particularly concerning long-term outcomes and the potential need for concurrent immunosuppressive therapy to optimize implant success, potentially by reducing ongoing inflammation around the implant and preserving residual cochlear function [7].

## Conclusions

This case highlights several important considerations. First, clinicians should add autoimmune etiologies as part of their differentials in patients presenting with progressive, unexplained SNHL, particularly when associated with neurological symptoms or systemic autoimmune disorders. Early diagnosis and timely initiation of immunotherapy may prevent irreversible auditory damage. Secondly, collaborative management involving neurology, otolaryngology, and audiology teams is crucial in addressing both the neurological and audiological components of autoimmune encephalitis. Finally, prospective studies are necessary to elucidate the pathophysiology of auditory involvement in LGI-1 encephalitis and evaluate the efficacy and safety of CI in this unique patient population. Long-term outcomes from CI in other autoimmune inner ear diseases may help inform early hypotheses and guide study design.
